# PARASYMPATHETIC INFLUENCE ON COGNITIVE AGING IS MODERATED BY SYMPATHETIC ACTIVITY, ESPECIALLY IN EARLY MIDLIFE

**DOI:** 10.1093/geroni/igz038.355

**Published:** 2019-11-08

**Authors:** Erik L Knight, Ryan Giuliano, Sean Shank, Megan Clarke, David M Almeida

**Affiliations:** 1 Pennsylvania State University, University Park, Pennsylvania, United States; 2 University of Manitoba, Manitoba, Winnipeg, Canada

## Abstract

The two branches of the autonomic nervous system (ANS) have been individually linked to age-related changes in cognitive functioning: The parasympathetic nervous system (PNS) is thought to support healthy cognitive aging, whereas the sympathetic nervous system (SNS) has been linked to heightened cognitive decline. Despite these separate findings and despite the integrative nature of the ANS, little work has examined the two branches simultaneously to better understand their interactive effects on age-related cognitive changes. We examined cognitive change in two waves of the MIDUS cognitive project and indexed PNS and SNS activity from heart rate variability and epinephrine levels (respectively) from the MIDUS biomarker project (n = 764, 56% female, mean age = 54.1 years). Our findings indicate that higher PNS levels attenuate cognitive decline, but only among individuals with low SNS levels; at higher SNS levels, the beneficial effects of the PNS are blocked. Further, lower PNS levels can be somewhat compensated for by increased SNS levels. This pattern was most robust among individuals transitioning to mid-life (i.e., 35-40 years old at the initial cognitive test). These results suggest that interventions targeting the ANS as a modifiable factor in cognitive aging should consider both ANS branch’s effects simultaneously, particularly in the early stages of midlife.

